# Small-Target Detection between SAR Images Based on Statistical Modeling of Log-Ratio Operator

**DOI:** 10.3390/s19061431

**Published:** 2019-03-23

**Authors:** Chao Chen, Kuihua Huang, Gui Gao

**Affiliations:** 1 College of System Engineering, National University of Defense Technology, Sanyi Avenue, Changsha 410073, China; chenc1997@nudt.edu.cn; 2 College of Traffic Engineering, Hunan University of Technology, Zhuzhou 412007, China; 3 Faculty of Geoscience and Environmental Engineering, Southwest Jiaotong University, Chengdu 611756, China

**Keywords:** SAR (synthetic aperture radar), image ratio, target detection, statistical modeling

## Abstract

The log-ratio (LR) operator is well suited for change detection in synthetic aperture radar (SAR) amplitude or intensity images. In applying the LR operator to change detection in multi-temporal SAR images, a crucial problem is how to develop precise models for the LR statistics. In this study, we first derive analytically the probability density function (PDF) of the LR operator. Subsequently, the PDF of the LR statistics is parameterized by three parameters, i.e., the number of looks, the coherence magnitude, and the true intensity ratio. Then, the maximum-likelihood (ML) estimates of parameters in the LR PDF are also derived. As an example, the proposed statistical model and corresponding ML estimation are used in an operational application, i.e., determining the constant false alarm rate (CFAR) detection thresholds for small target detection between SAR images. The effectiveness of the proposed model and corresponding ML estimation are verified by applying them to measured multi-temporal SAR images, and comparing the results to the well-known generalized Gaussian (GG) distribution; the usefulness of the proposed LR PDF for small target detection is also shown.

## 1. Introduction

During the last two decades, the problem of change detection [1] from at least two geo-registered synthetic aperture radar (SAR) images, collected at different times, has been recognized to be important for practical applications related to environmental monitoring [2,3,4], damage assessment [5,6], urban studies [7,8], and forest monitoring [9,10,11]. Many approaches [12,13] have been developed in the literature to deal with the problem of change detection. Based on these approaches, a brief and excellent review of state-of-the-art change detection techniques of SAR images has been given in [14]. Additionally, more recent methods, for instance, based on nonlocal means of denoising, mean-shift clustering, and multiple neural-network models, as well as a hierarchical method, have been reported in [7,14,15,16], respectively.

Specifically, as one of the most important applications of change detection technology, the identification of small objects (i.e., objects occupying only a few pixels, such as vehicles) between two SAR amplitude or intensity images has also been discussed extensively [17,18,19], where small objects only appear in one of two SAR images, and are labelled as a changed class in a fixed scene.

Because of the multiplicative nature of speckle noise [1,20], it has been proven to be more effective to use the ratio operator than the difference operator [21,22] as a change-detection metric to compare two SAR temporal images [23,24,25]. In practice, to uncompress the range of variations of the image ratio, and to ensure the model adequacy for change detection purposes, the log-ratio (LR) operator is preferred [13,24]. To obtain the changed and unchanged pixels between two SAR images, the most popular way to use the LR operator is to employ statistical approaches by linking the distribution model of the LR statistics and a specific threshold decision technique, e.g., the expectation maximization (EM) [26] and Kittler–Illingworth (KI) threshold methods [27,28,29,30]. In the case of small objects, since the changed small objects occupy only very few pixels with respect to the large background, they almost do not influence the statistical characteristics of the whole image, facilitating the decision of determining changed and unchanged classes using a simple, adaptive threshold technique, e.g., the constant false alarm rate (CFAR) [20], if only the distribution model of LR statistics for the background can be derived. Hence, the statistical modeling of LR statistics for the unchanged background in images acquired at different times is first needed for this purpose. A relevant study was carried out [31,32], where a statistical distribution of ratio operators for images of two polarization or interferometry channels was developed. Several investigations have shown that this model was also appropriate to describe the statistical behaviors of the ratio operator between two SAR images with nominally the same sensor setup (frequency, polarization, and illumination geometry) and identical processing parameters (e.g., multi-look processing) due to the same rationale for multi-channel images [17,20]. However, an appropriate theoretical probability density function (PDF) of LR statistics and parameter estimates have not been given in the literature to date. Instead, a generalized Gaussian (GG) PDF has been widely used to empirically model the LR statistics [3,23,26,27,28,33].

The objective of this paper is to address the aforementioned issues with respect to the characterization of LR statistics and the corresponding parameter estimates. The contributions in this paper can be summarized as follows.

1. By transforming the image ratio into the log-scale domain, we derive the PDF of the LR statistics. Moreover, ML estimates [34] of the parameters in the LR PDF are derived analytically.
2. The change detection of vehicles based on the measured data is assessed by combining the proposed parameter estimation and the CFAR technique. As a result, the usefulness of the proposed distribution model and the effectiveness of the corresponding parameter estimation are verified for small target detection.

The rest of this paper is organized as follows. In Section 2, the detailed derivations of the proposed model and ML parameter estimation of the LR statistics are given. Section 3 is devoted to the applications of the proposed model for small target detection; the experimental analysis of typical measured data is also given in this section. Section 4 concludes the paper.

## 2. Modeling of LR Statistics

### 2.1. LR Statistics and Distribution

Let us consider two equal-sized and co-registered SAR intensity images with M rows and N columns, $I_{1}=\left\{I_{1}\left(i,j\right),1\leq i\leq M,1\leq j\leq N\right\}$ (reference image) and $I_{2}=\left\{I_{2}\left(i,j\right),1\leq i\leq M,1\leq j\leq N\right\}$ (test image), acquired over the same geographical region at different times. The ratio of image intensity can be defined as the ratio of average intensity computed for a window of size w×w, as follows:

(1) $$R\left(i,j\right)=\frac{\sum_{\left(k,l\right)\in \Omega_{i,j}}I_{2}\left(k,l\right)}{\sum_{\left(k,l\right)\in \Omega_{i,j}}I_{1}\left(k,l\right)}$$

where $\Omega_{i,j}$ defines the neighbourhood around pixel (i,j) in a sliding window of size w×w. 

Furthermore, for a speckled, but otherwise homogeneous region, it is well-known that the statistics of the intensity ratio as shown in Equation (1) can be characterized by the density [31,32]

(2) $$p_{R}\left(r\right)=\frac{\tau^{n}\Gamma \left(2n\right){\left(1-\rho^{2}\right)}^{n}}{\Gamma \left(n\right)\Gamma \left(n\right)}\frac{\left(\tau +r\right)r^{n-1}}{{\left({\left(\tau +r\right)}^{2}-4\tau \rho^{2}r\right)}^{\left(2n+1\right)/2}},\;r\in {\mathbb{R}}^{+};\tau ,n\text{>}0,\;\mathrm{and}\;0\leq \rho \leq 1$$

where n is the number of looks, and Γ is the Gamma function. Equation (2) is still valid in the presence of texture, provided that the backscattering coefficient with one n-look pixel is constant [17,32]. The parameter ρ represents the coherence magnitude between the two images. The symbol τ denotes the true intensity ratio, which can be estimated using [31]

(3) $$\tau =\frac{E\left(I_{2}\right)}{E\left(I_{1}\right)}$$

where E(·) is the expectation. It should be noted that the expectations in Equation (3) are computed by averaging over each entire image. By transforming the intensity ratio into the log-scale domain, the PDF of the LR statistics X, X=logR, can be derived using Equation (2) to give (see Appendix A)

(4) $$p_{X}\left(x\right)=\frac{\tau^{n}\Gamma \left(2n\right){\left(1-\rho^{2}\right)}^{n}}{\Gamma \left(n\right)\Gamma \left(n\right)}\frac{\left(\tau +e^{x}\right)e^{x}{}^{n}}{{\left({\left(\tau +e^{x}\right)}^{2}-4\tau \rho^{2}e^{x}\right)}^{\left(2n+1\right)/2}},\;x\in \mathbb{R};\tau ,n\text{>}0,\;\mathrm{and}\;0\leq \rho \leq 1$$

**Figure 1:**
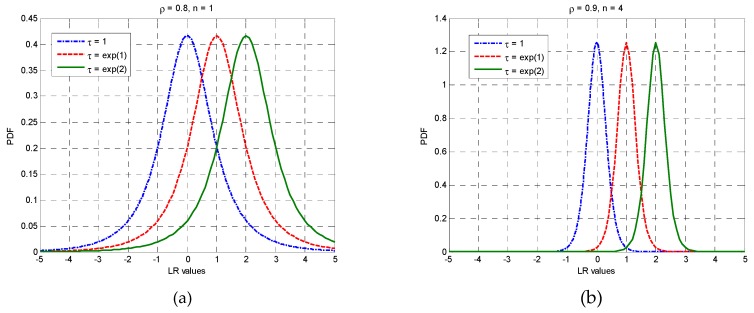
Theoretical probability density functions (PDFs) of log-ratio (LR) statistics with varying parameters: (**a**) fixed n=1 and ρ=0.8 for different values of τ; (**b**) fixed n=4 and ρ=0.9 for different values of τ.

As shown in Figure 1, the PDF defined by (4) has the desirable characteristic of being symmetrical about ln(τ). This can be proved mathematically as follows. 

Assuming that two arbitrary variables, $x_{1}$ and $x_{2}$, are symmetrical about ln(τ), then $x_{1}=\ln\left(\tau \right)+a$ and $x_{2}=\ln\left(\tau \right)-a$, where a is a nonzero constant. Via (4), the PDF value of $x_{1}$ is

(5) $$\begin{array}{ll} p_{X}\left(x_{1}\right) & =\frac{\tau^{n}\Gamma \left(2n\right){\left(1-\rho^{2}\right)}^{n}}{\Gamma \left(n\right)\Gamma \left(n\right)}\frac{\left(\tau +e^{\ln\left(\tau \right)+a}\right)e^{n\ln\left(\tau \right)+na}}{{\left({\left(\tau +e^{\ln\left(\tau \right)+a}\right)}^{2}-4\tau \rho^{2}e^{\ln\left(\tau \right)+a}\right)}^{\left(2n+1\right)/2}}\\ & =\frac{\Gamma \left(2n\right){\left(1-\rho^{2}\right)}^{n}}{\Gamma \left(n\right)\Gamma \left(n\right)}\frac{\left(1+e^{a}\right)e^{na}}{{\left({\left(1+e^{a}\right)}^{2}-4\rho^{2}e^{a}\right)}^{\left(2n+1\right)/2}} \end{array}$$

Likewise, $p_{X}\left(x_{2}\right)$ can be written as

(6) $$p_{X}\left(x_{2}\right)=\frac{\Gamma \left(2n\right){\left(1-\rho^{2}\right)}^{n}}{\Gamma \left(n\right)\Gamma \left(n\right)}\frac{\left(1+e^{-a}\right)e^{-na}}{{\left({\left(1+e^{-a}\right)}^{2}-4\rho^{2}e^{-a}\right)}^{\left(2n+1\right)/2}}$$

By multiplying the numerator and denominator of the right-hand side in Equation (5) by the same factor $e^{-\left(2n+1\right)a}$, we obtain

(7) $$\begin{array}{ll} p_{X}\left(x_{1}\right) & =\frac{\Gamma \left(2n\right){\left(1-\rho^{2}\right)}^{n}}{\Gamma \left(n\right)\Gamma \left(n\right)}\frac{\left(1+e^{a}\right)e^{na}e^{-\left(2n+1\right)a}}{e^{-\left(2n+1\right)a}{\left({\left(1+e^{a}\right)}^{2}-4\rho^{2}e^{a}\right)}^{\left(2n+1\right)/2}}\\ & =\frac{\Gamma \left(2n\right){\left(1-\rho^{2}\right)}^{n}}{\Gamma \left(n\right)\Gamma \left(n\right)}\frac{\left(1+e^{-a}\right)e^{-na}}{{\left(e^{-2a}{\left(1+e^{a}\right)}^{2}-4\rho^{2}e^{a}e^{-2a}\right)}^{\left(2n+1\right)/2}} \end{array}$$

As seen from (7), the LR PDF shown in (4) is symmetrical around ln(τ). This symmetry is rather important for the simplification of change detection problems in practice. As is known, there might be positive and negative changed parts among the changed pixels [20,23,28]. Taking the small target detection as an example, we often do not know in advance what targets appear in which of the two images (sometimes the changed targets appear in both co-registered SAR images), thus it cannot be determined whether the target pixels should correspond to the positive or negative changed parts after LR operation. As a result, two thresholds are commonly needed to classify the changed and unchanged categories. However, the number of decision thresholds might decrease to one if it is assumed that the positive and negative changed parts are identically important, because of the symmetrical characteristic of the LR PDF.

### 2.2. ML Estimation

As shown in Equation (4), the PDF of random variable X is parameterized by three parameters, τ, n, and ρ, in which τ is easy to obtain by Equation (3). If τ is estimated by Equation (3) in advance, it can be regarded as known. Therefore, the central task of parameter estimation for the density shown in Equation (4) is focused on the estimates of the number of looks n, and the coherence magnitude ρ. Generally, the ML approach, the method of moments (MoM) [34], as well as the more recent method of log-cumulants (MoLC) [12,28,35], could be candidates for the parameter estimates of a known density. However, an analytical description of the moments of the LR statistics is not easy to acquire by Equation (4), if not impossible, thus MoM and MoLC are not realistic for estimating parameters n and ρ. The ML estimates of n and ρ are derived in the following.

For a sample set $X_{1},X_{2},\cdots ,X_{m}$ of m independent observations of the random variable X, all with the same distribution obeying Equation (4), the log-likelihood function can be derived as

(8) $$\begin{array}{ll} l\left(n,\rho \right) & =\ln\left(\overset{m}{\underset{i=1}{\Pi }}p_{X}\left(x_{i}\right)\right)=\sum_{i=1}^{m}\ln p_{X}\left(x_{i}\right)\\ & =nm\ln\tau +nm\ln\left(1-\rho^{2}\right)+m\ln\left(\Gamma \left(2n\right)\right)-2m\ln\left(\Gamma \left(n\right)\right)\\ & \;+\sum_{i=1}^{m}\ln\left(\tau +e^{X_{i}}\right)+n\sum_{i=1}^{m}X_{i}-\frac{2n+1}{2}\sum_{i=1}^{m}\ln\left({\left(\tau +e^{X_{i}}\right)}^{2}-4\tau \rho^{2}e^{X_{i}}\right) \end{array}$$

Hence, the ML estimates $\hat{n}$ and $\hat{\rho }$ correspond to parameters n and ρ, respectively, and can be written as

(9) $$l\left(\hat{n},\hat{\rho }\right)=\underset{n,\rho }{\max}\;l\left(n,\rho \right)$$

The previous maximization can be realized by applying the first-order derivatives of the log-likelihood function shown in Equation (8) with respect to n and ρ. Equating these derivatives to zero, we obtain the nonlinear equations as follows:

(10) $$\left\{ \begin{array}{l} \Psi \left(\hat{n}\right)-\Psi \left(2\hat{n}\right)=\frac{\ln\tau }{2}+\frac{\ln\left(1-{\hat{\rho }}^{2}\right)}{2}+\frac{\sum_{i=1}^{m}X_{i}-\sum_{i=1}^{m}\ln\left({\left(\tau +e^{X_{i}}\right)}^{2}-4\tau {\hat{\rho }}^{2}e^{X_{i}}\right)}{2m}\\ \sum_{i=1}^{m}\frac{\left(1-{\hat{\rho }}^{2}\right)e^{X_{i}}}{{\left(\tau +e^{X_{i}}\right)}^{2}-4\tau {\hat{\rho }}^{2}e^{X_{i}}}=\frac{\hat{n}m}{2\tau \left(2\hat{n}+1\right)} \end{array}\right.$$

where Ψ(·) is the so-called Psi or Digamma function [34], i.e., the logarithmic derivative of the Gamma function. The solution of the above equations corresponds to the ML estimation of the parameters n and ρ. 

## 3. Applications to Small target Detection

In this section, we test our proposed distribution model and its parameter estimation approach by applying it to small target detection using measured multi-temporal SAR data. Our goals are to gauge the performance of the proposed distribution model of LR statistics and corresponding parameter estimation, and to determine the usefulness of the proposed distribution model for small target detection. For these purposes, a dataset of VHF-band CARABAS-II SAR images containing changed vehicles is used to test the techniques.

### 3.1. Data Description

**Figure 2:**
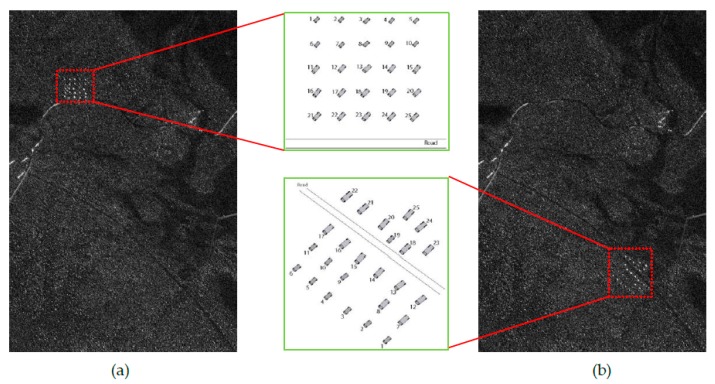
VHF-band synthetic aperture radar (SAR) image pair (vehicles are located in red boxes) used to the first experiment: (**a**) reference image of pass 5 in mission 2; (**b**) test image of pass 5 in mission 4. Note that the sketch of target deployment is also given in this figure.

**Figure 3:**
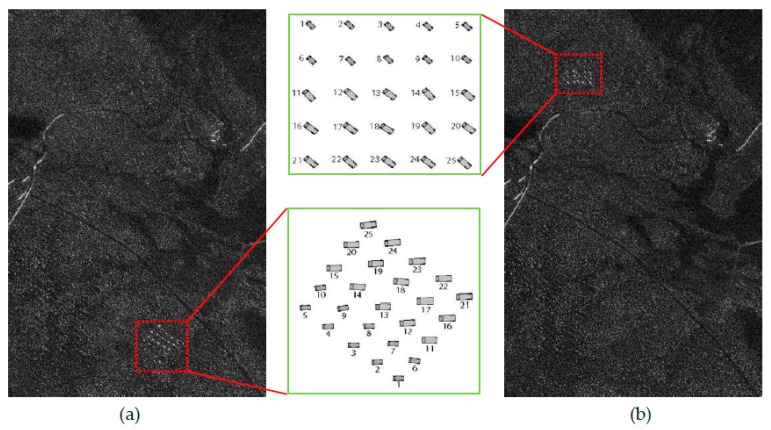
VHF-band SAR image pair (vehicles are located in red boxes) used to the second experiment: (**a**) reference image of pass 3 in mission 5; (**b**) test image of pass 3 in mission 3. Note that the sketch of target deployment is also given in this figure.

The 24 public SAR images included in VHF-band CARABAS-II dataset [18] during a flight campaign in northern Sweden in 2002 are used to validate the proposed model and its potential for small target detection. These images were collected by four vehicle deployments numbered as mission 2, 3, 4, and 5. For each of the four missions, six images with different passes numbered 1 to 6 are available. Each image contains 25 vehicles of three types concealed by foliage, with a pixel spacing of 1 m × 1 m (a nominal resolution of 2.5 m × 2.5 m) and a size of 3000 rows by 2000 columns. Two arbitrary images (one as the reference image, the other as the test image) with the different missions but the same pass can be used to form an image pair serving as the detection vehicle target. Herein, we report the fitting and detection results of two typical image pairs; other test results are consistent with these reported ones. As examples, Figure 2 and Figure 3 show two image pairs from the repeated pass, but different missions.

### 3.2. Model Fitting 

We test the proposed model and parameter estimation by applying it to the data described above. It is worth noting that the distribution of the LR image is expected to be close to Gaussian [33,36]. However, several studies, e.g., [28,33], have shown that a Gaussian approximation is not sufficiently accurate to characterize the LR statistics in practice. Instead, a more general and accurate parametric model, i.e., the GG model, was adopted to describe the statistical behavior of the LR images. Although the GG model is still empirical, it is an attractive candidate for modeling LR images, and has proven to be useful for change detection in SAR images [3,28,33]. Hence, to assess the modeling capability of the proposed model, we compare it with the GG model in this investigation. The GG distribution is characterized by the following PDF [37,38]

(11) $$p_{X}\left(x\right)=\frac{\gamma c}{2\Gamma \left(1/c\right)}\exp\left[-{\left|\gamma \left(x-\mu \right)\right|}^{c}\right],\;\;x,\mu \in \mathbb{R};\gamma ,c\text{>}0$$

where $\gamma =\frac{1}{\sigma }\sqrt{\Gamma \left(\frac{3}{c}\right)/\Gamma \left(\frac{1}{c}\right)}$, in which σ is the variance of the distribution. The parameters μ and c are the mean and shape parameters of the distribution, respectively. The symbol |⋅| represents the absolute value. It should be emphasized that c=2 yields the Gaussian density function, which indicates that the GG model encompasses the modeling ability of the Gaussian distribution. Besides, the GG PDF shown in Equation (11) is symmetrical around μ. The parameter estimation technique of the GG model can be found in [37].

Similar to conventional change detection applications, such as environmental monitoring [2,3,4] and damage assessment [5], speckle filtering with a finite-sized window on a SAR image pair is necessary before calculating the LR statistics for small target detection applications, because the presence of speckle can lead to poor performance of the detection algorithm [28,39]. However, an appropriate despeckling window size is not easy to determine, and has to be obtained empirically because no prior training pixels are available. As suggested in [18,19], a 5 × 5 window is feasible for the detection of small targets when the resolution of the SAR image is not larger than 1 m, because it represents a good compromise between speckle smoothing and detection of small targets. In addition, it is worth noting that speckle filtering is less important at the model fitting stage than at the small target detection stage, based on the fact that the theoretical models should accurately describe the LR statistics for any finite size of filtering window. For brevity, we report results using window sizes of 1 × 1 (i.e., non-filtered) and 5 × 5 at the model fitting stage as typical examples; the results of other window sizes are consistent with these two window sizes. Correspondingly, at the small target detection stage, the detection results are reported for both previous experiments only when the neighbourhood Ω in Equation (1) is taken with a 5 × 5 window before calculating the LR statistics, as discussed in Section 3.3. 

**Figure 4:**
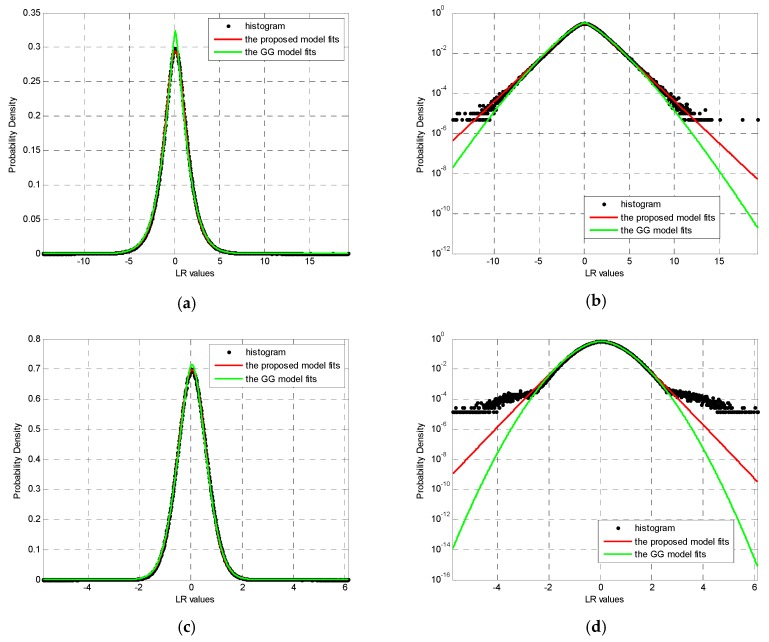
Fitting results of the proposed model for the data shown in Figure 2: (**a**,**b**) are the results for a 1 × 1 filtering window displayed on linear and semi-log scales, respectively; (**c**,**d**) are the results for a 5 × 5 filtering window displayed on linear and semi-log scales, respectively.

**Figure 5:**
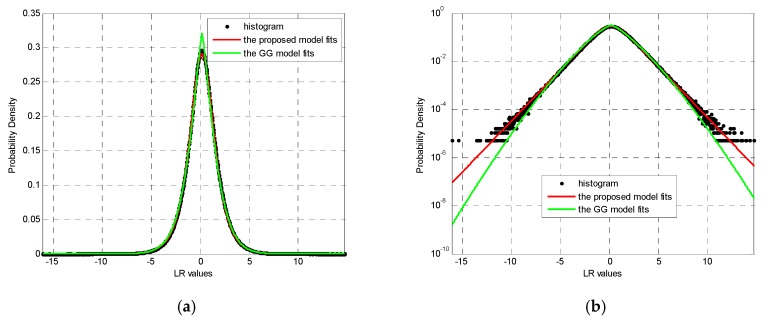

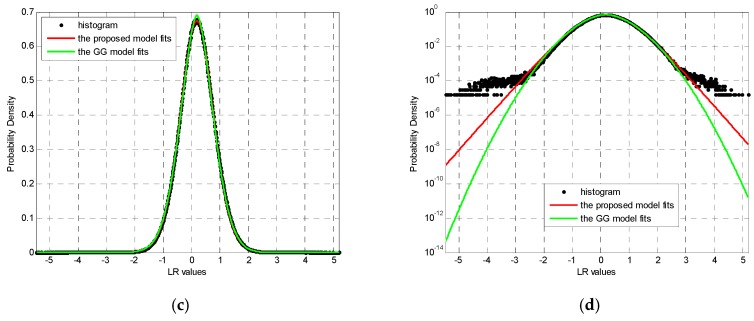
Fitting results of the proposed model for the data shown in Figure 3: (**a**,**b**) are the results for a 1 × 1 filtering window displayed on linear and semi-log scales, respectively; (**c**,**d**) are the results for a 5 × 5 filtering window displayed on linear and semi-log scales, respectively.

The parameter estimates of the proposed model and the GG model for two experiments with different window sizes are listed in Table 1. Based on these estimates, the fitting results are provided so that the performance difference in fitting the whole histogram and the histogram tails can be seen clearly (Figure 4 and Figure 5). To clearly show the details of the fitting results, all figures are plotted both on linear and semi-log scales. It is clear from a visual inspection of these figures that almost perfect fitting results are obtained by the theoretical model presented in Equation (4) for the two SAR LR images. It is also clear, from a visual comparison between the estimated PDFs and image histograms, that the proposed model in Equation (4) agrees better with the given LR statistics than the GG model, despite the filtering window sizes, indicating the high precision of the fitting LR statistics using the proposed model. Furthermore, Figure 4b,d, along with Figure 5b,d, show less deviation from the tail of the histograms using the proposed model compared to using the GG model, implying better detection performance of the proposed model, because the histogram tail commonly contributes to false alarms.

To quantitatively assess the fitting results and compare the different models, specific criteria as goodness-of-fit measures can be employed. In information theory, the Kullback–Leibler (KL) divergence is known to be an effective measure and can realize the global comparison of PDFs [40].

Given the theoretical PDF p(w), and observed PDF q(w), the KL divergence or relative entropy between two densities is defined as [40]:

(12) $$D\left(q||p\right)=\int q\left(w\right)\log_{2}\left(\frac{q\left(w\right)}{p\left(w\right)}\right)dw$$

The approximated numerical calculation is given by

(13) $$D\left(q||p\right)=\sum q\left(w\right)\Delta w\cdot \log_{2}\left(\frac{q\left(w\right)\Delta w}{p\left(w\right)\Delta w}\right)=\sum Q\left(w\right)\cdot \log_{2}\left(\frac{Q\left(w\right)}{P\left(w\right)}\right)$$

where Q(w) is the value of probability of the observeddata, and P(w) denotes the theoretical probability value. Note that D(q||p) is not symmetrical (i.e., D(q||p)≠D(p||q)). In our case, the symmetrized KL divergence is adopted as

(14) $$D_{KL}=D\left(q||p\right)+D\left(p||q\right)$$

When the observed density equals the theoretical density, $D_{KL}$ is zero. Otherwise, $D_{KL}$ is a positive value. The KL divergence reflects the overall similarity of the observed and theoretical densities. The smaller the value of KL divergence, the higher the similarity, indicating better fitting accuracy.

Based on Equation (14), the KL values of the fitting results shown in Figure 4 and Figure 5 are compared in Table 2. This table clearly confirms the conclusion that were drawn by visual inspection, i.e., the proposed model is in better agreement with the histograms of the LR statistics for different window sizes than with the GG model. This is to be expected, because the KL values are smaller for the proposed model than for the GG model. In summary, the proposed model shown in Equation (4) has better accuracy in describing the statistical behaviors of the LR operator and is superior to the known GG model. Moreover, the corresponding ML estimators of parameters are also effective. Additionally, unlike the empirical GG model, the proposed model is rigorously derived based on mathematics and SAR principles, making it more appropriate and controllable in practical applications.

### 3.3. Detection Application

For change detection problems, a popular decision technique is the Kittler–Illingworth (KI) unsupervised threshold selection criterion which has been developed under the Gaussian, GG, Nakagami, Log-Normal, and Weibull assumptions for modeling the statistical distributions of changed and unchanged classes in either the LR or ratio images [28,33]. As stated in [23,28], the KI method takes change detection in the ratio or LR images as a two-category classification problem, and performs well on the condition that the changed and unchanged parts have comparable histogram peaks. However, in small target detection, this condition generally cannot be satisfied because small targets only comprise a few changed pixels in a SAR image pair, leading to a monomodal LR image histogram, and the targets labelled as changed pixels fall into the tail part of the histogram. As a result, the KI method can result in several detection errors [23] and cannot be used for small target detection. 

In contrast, the CFAR technique is well-known because of its adaptive ability of determining a threshold in the field of small target detection. As for change detection, because there might be positive and negative changed parts among the change pixels, two thresholds $T_{1}$ and $T_{2}$ (assuming $T_{1}>T_{2}$) are often needed in practice. It is also reasonable to assume that the positive and negative changed parts contribute the same false alarm rate, when one does not know in advance what targets will appear in which of the two images. 

Under a given value of false alarm probability, denoted by $P_{fa}$, the corresponding CFAR thresholds for a specific density $p_{X}\left(x\right)$ is obtained from

(15) $$\begin{array}{ll} P_{fa} & =\int_{T_{1}}^{\infty }p_{X}\left(x\right)dx+\int_{-\infty }^{T_{2}}p_{X}\left(x\right)dx\\ & =2\int_{T_{1}}^{\infty }p_{X}\left(x\right)dx \end{array}$$

Based on Equations (4) and (11), the threshold $T_{1}$ under different background assumptions (i.e., obeying the proposed model or being GG-distributed) can be accurately calculated by Equation (15) with the help of the numerical solution. Because of the symmetry of the two densities, $T_{1}$ and $T_{2}$ should be symmetrical about ln(τ) for the proposed model, and about μ for the GG distribution. Then, $T_{2}=2\ln\tau -T_{1}$ in the assumption of the proposed model, and $T_{2}=2\mu -T_{1}$ in the assumption of the GG distribution. Accordingly, for the test cell η in the LR image, the decision rule of detection can be adopted as

(16) $$\{\begin{array}{cc} \mathrm{target}: & \eta >T_{1}\;\mathrm{or}\;\eta <T_{2}\\ \mathrm{background}: & otherwise \end{array}$$

**Figure 6:**
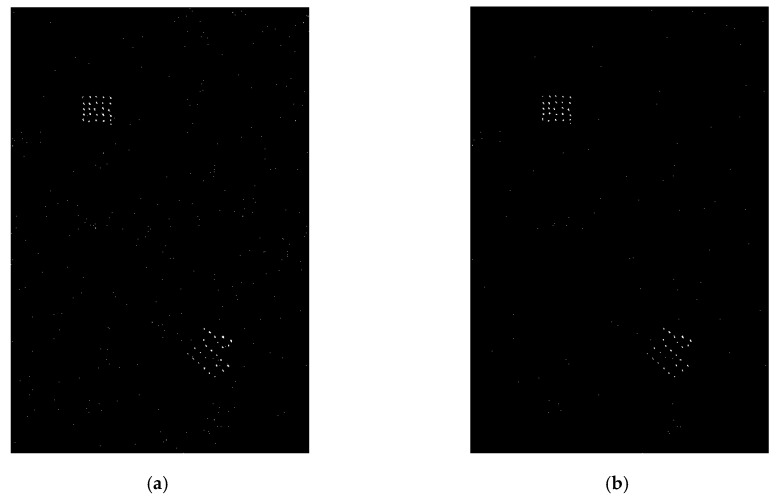
Constant false alarm rate (CFAR) detection results for the image pair shown in Figure 2: (**a**) detection results based on GG model; (**b**) detection results based on proposed model.

**Figure 7:**
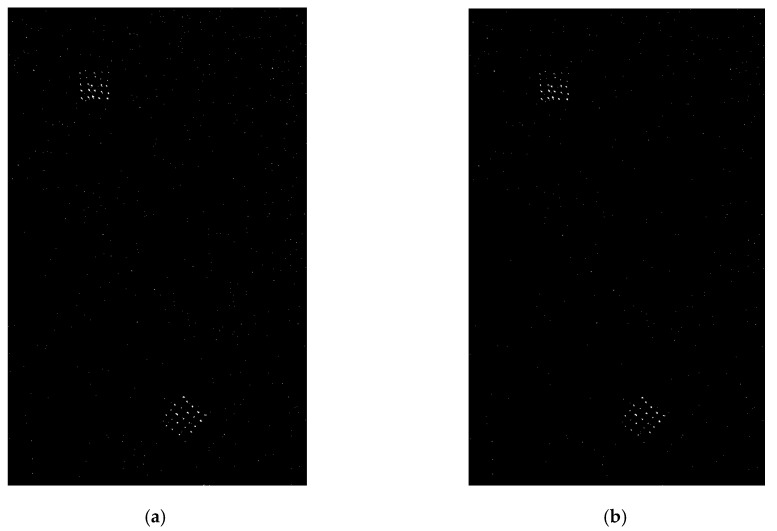
CFAR detection results for the image pair shown in Figure 3: (**a**) detection results based on GG model; (**b**) detection results based on proposed model.

Given the theoretical false alarm probability $P_{fa}=10^{-3}$, using (16) for the image pairs shown in Figure 2 and Figure 3, the corresponding CFAR detection results of the LR operator with the distribution assumptions of the proposed model and the GG model are given in Figure 6 and Figure 7, respectively.

First, Figure 6b and Figure 7b show that all targets are well detected by the LR CFAR detector based on the proposed model, and far fewer false alarms occur compared to CFAR detection using the GG model from a visual inspection. This demonstrates the effectiveness of the CFAR method based on the proposed model and allows us to complete the adaptive detection of small targets with a desirable false alarm probability, as expected. 

Second, if detections are found to exist within a radius of 10 m from a ground truth position, the target is declared to be found, and can be counted. All other detected changes that are not considered to be related to a target are regarded as false alarms. A whole performance comparison between the CFAR detections based on the proposed model and the GG model is provided in Table 3. In Table 3, the actual false alarm rate (FAR) is estimated by

(17) $$\mathrm{FAR}=\frac{N_{c}}{M\times N-N_{t}}$$

where $N_{c}$ is the number of false alarm pixels, M×N is the size of the detected image, and $N_{t}$ is the number of the detected target pixels.

From Table 3, on one hand, the actual FARs generated by both the GG model and proposed model do not rigorously match the theoretical $P_{fa}$. This is mainly due to that both the GG model and proposed model exhibit some deviation from the measured histogram tails, as shown in Figure 4d and Figure 5d. On the other hand, it can be seen that both the CFAR methods based on the GG model and the proposed model are able to detect all targets easily, but the detection method using the GG model generates higher FAR than the proposed model in two experiments. In other words, in terms of actual FAR, the CFAR method based on the proposed model shows better target detection performance than the CFAR method based the GG model. This is in accord with the analysis in Section 3.2, since the proposed model exhibits higher modeling capacity of LR statistics than the empirical GG model as a whole, as well as the tail part of the LR histogram, which contributes to most of the false alarms. In summary, all the results above suggest that the proposed LR PDF and its parameter estimation approach are promising for application in the detection of small targets between SAR images.

## 4. Conclusions

In this paper, we analytically derived the PDF of the LR operator, along with the ML estimates of parameters in the LR PDF. The experimental results using real data validate the effectiveness of the proposed model and ML estimation. Moreover, in combination with a commonly used CFAR decision technique, the proposed model and corresponding parameter estimation have excellent potential for applications in small target detection between SAR images. We expect to test more measured data in the future.

## Figures and Tables

**Table 1:** Parameter estimations with different window sizes. GG: generalized Gaussian.

Experiment No.	Window Size	GG Model $\left(\hat{\mu },\hat{\sigma },\hat{c}\right)$	Proposed Model $\left(\hat{\tau },\hat{n},\hat{\rho }\right)$
1	1 × 1	(0.0455, 1.6568, 1.2908)	(1.0465, 0.9714, 0.5514)
	5 × 5	(0.0429, 0.6047, 1.7263)	(1.0439, 4.1180, 0.6049)
2	1 × 1	(0.1893, 1.6671, 1.3012)	(1.2083, 0.9731, 0.5357)
	5 × 5	(0.1852, 0.6201, 1.7589)	(1.2034, 4.3307, 0.5333)

Note: $\hat{\mu }$, $\hat{\sigma }$ and $\hat{c}$ are estimates of parameters μ, σ and c in Equation (11), respectively; $\hat{\tau }$, $\hat{n}$ and $\hat{\rho }$ are estimates of parameters τ, n and ρ in Equation (4), respectively.

**Table 2:** Comparison of fitting accuracies.

Experiment No.	Window Size	$D_{KL}$	
		GG Model	Proposed Model
**1**	1 × 1	0.0015	0.0003
	5 × 5	0.0024	0.0013
2	1 × 1	0.0016	0.0004
	5 × 5	0.0014	0.0008

**Table 3:** Whole Detection Performance Comparison with $P_{fa}=10^{-3}$. FAR: false alarm rate.

Experiment No.	Number of Targets	Number of Detected Targets		Actual FAR	
		GG Model	Proposed Model	GG Model	Proposed Model
1	50	50	50	0.7064 × 10^−3^	0.2230 × 10^−3^
2	50	50	50	0.8113 × 10^−3^	0.4956 × 10^−3^

## Appendix A

Because X=logR, $R=e^{X}$. Hence, Jacobiandet |J| can be derived as $\left|J\right|=\left|\partial R/\partial X\right|=e^{X}$. Basedon (2) and utilizing the transform $R=e^{X}$, thePDF of random variable X can be given as

(A1) $$\begin{array}{ll} p_{X}\left(x\right) & =\left|J\right|\frac{\tau^{n}\Gamma \left(2n\right){\left(1-\rho^{2}\right)}^{n}}{\Gamma \left(n\right)\Gamma \left(n\right)}\frac{\left(\tau +e^{x}\right)e^{x}{}^{n-x}}{{\left({\left(\tau +e^{x}\right)}^{2}-4\tau \rho^{2}e^{x}\right)}^{\left(2n+1\right)/2}}\\ & =e^{x}\frac{\tau^{n}\Gamma \left(2n\right){\left(1-\rho^{2}\right)}^{n}}{\Gamma \left(n\right)\Gamma \left(n\right)}\frac{\left(\tau +e^{x}\right)e^{x}{}^{n-x}}{{\left({\left(\tau +e^{x}\right)}^{2}-4\tau \rho^{2}e^{x}\right)}^{\left(2n+1\right)/2}}\\ & =\frac{\tau^{n}\Gamma \left(2n\right){\left(1-\rho^{2}\right)}^{n}}{\Gamma \left(n\right)\Gamma \left(n\right)}\frac{\left(\tau +e^{x}\right)e^{x}{}^{n}}{{\left({\left(\tau +e^{x}\right)}^{2}-4\tau \rho^{2}e^{x}\right)}^{\left(2n+1\right)/2}} \end{array}$$

## References

[1] White R.G. (1990). Change detection in SAR imagery. Int. J. Remote Sens..

[2] Liew S.C., Kam S.P., Tuong T.P., Chen P., Minh V.Q., Lim H. (1998). Application of multitemporal ERS-1 synthetic aperture radar in delineating rice cropping systems in the Mekong River Delta, Vietnam. IEEE Trans. Geosci. Remote Sens..

[3] Martinis S., Twele A., Voigt S. (2011). Unsupervised extraction of flood-induced backscatter changes in SAR data using Markov modeling on irregular graphs. IEEE Trans. Geosci. Remote Sens..

[4] Dellepiane S.G., Angiati E. (2012). A new method for cross-normalization and multitemporal visualization of SAR images for detection of flooded areas. IEEE Trans. Geosci. Remote Sens..

[5] Bovolo F., Bruzzone L. (2007). A split-based approach to unsupervised change detection in large-sized multitemporal images: Application to Tsunami-damage assessment. IEEE Trans. Geosci. Remote Sens..

[6] Dekker R.J. (2011). High-resolution radar damage assessment after the earthquake in Haiti on 12 January 2010. IEEE J. Sel. Top. Appl. Earth Observ. Remote Sens..

[7] Yousif O., Ban Y. (2013). Improving urban change detection from multitemporal SAR images using PCA-NLM. IEEE Trans. Geosci. Remote Sens..

[8] Ban Y., Yousif O. (2012). Multitemporal spaceborne SAR data for urban change detection in China. IEEE J. Sel. Top. Appl. Earth Observ. Remote Sens..

[9] Hame T., Heiler I., Miguel-Ayanz J.S. (1998). An unsupervised change detection and recognition system for forestry. Int. J. Remote Sens..

[10] Grover K., Quegan S., da Costa Fretias C. (1999). Quantitative estimation of tropical forest cover by SAR. IEEE Trans. Geosci. Remote Sens..

[11] Quegan S., Le Toan T., Yu J.J., Ribbes F., Floury N. (2000). Multitemporal ERS SAR analysis applied to forest mapping. IEEE Trans. Geosci. Remote Sens..

[12] Chatelain F., Tourneret J.-Y., Inglada J. (2008). Change detection in multisensorSAR images using bivariate Gamma distribution. IEEE Trans. Image Process..

[13] Gong M., Cao Y., Wu Q. (2012). A neighborhood-based ratio approach for change detection in SAR images. IEEE Geosci. Remote Sens. Lett..

[14] Aiazzi B., Alparone L., Baronti S., Garzelli A., Zoppetti C. (2013). Nonparametric change detection in multitemporal SAR images based on Mean-Shift clustering. IEEE Trans. Geosci. Remote Sens..

[15] Pratola C., Frate F.D., Schiavon G., Solimini D. (2013). Toward fully automatic detection of changes in suburban areas from VHR SAR images by combining multiple Neural-Network models. IEEE Trans. Geosci. Remote Sens..

[16] Bovolo F., Bruzzone L. (2013). A hierarchical approach to change detection in very high resolution SAR images for surveillance applications. IEEE Trans. Geosci. Remote Sens..

[17] Dierking W., Skriver H. (2002). Change detection for thematic mapping by means of airborne multitemporal polarimetric SAR imagery. IEEE Trans. Geosci. Remote Sens..

[18] Lundberg M., Ulander L.M.H., Pierson W., Gustavsson A. (2006). A challenge problem for detection of targets in foliage. Proc. Algorithm Synth. Aperture Radar Imag. XIII.

[19] Gao G., Wang X., Niu M., Zhou S. (2014). Modified log-ratio operator for change detection of synthetic aperture radar targets in forest concealment. J. Appl. Remote Sens..

[20] Oliver C.J., Quegan S. (1998). Understanding Synthetic Aperture Radar Images.

[21] Bruzzone L., Prieto D.F. (2000). Automatic analysis of the difference image for unsupervised change detection. IEEE Trans. Geosci. Remote Sens..

[22] Bovolo F., Bruzzone L. (2007). A theoretical framework for unsupervised change detection based on change vector analysis in the polar domain. IEEE Trans. Geosci. Remote Sens..

[23] Xiong B., Chen Q., Jiang Y., Kuang G. (2012). A threshold selection method using two SAR change detection measures based on the Markov random field model. IEEE Geosci. Remote Sens. Lett..

[24] Carincotte C., Derrode S., Bourennane S. (2006). Unsupervised change detection on SAR images using fuzzy hidden Markov chains. IEEE Trans. Geosci. Remote Sens..

[25] Rignot E.J.M., Van Zyl J.J. (1993). Change detection techniques for ERS-1 SAR data. IEEE Trans. Geosci. Remote Sens..

[26] Bazi Y., Bruzzone L., Melgani F. (2007). Image thresholding based on the EM algorithm and the generalized Gaussian distribution. Pattern Recognit..

[27] Bazi Y., Bruzzone L., Melgani F. (2006). Automatic identification of the number and values of decision thresholds in the log-ratio image for change detection in SAR images. IEEE Geosci. Remote Sens. Lett..

[28] Moser G., Serpico B. (2006). Generalized minimum-error thresholding for unsupervised change detection from SAR amplitude imagery. IEEE Trans. Geosci. Remote Sens..

[29] Kittler J., Illingworth J. (1986). Minimum error thresholding. Pattern Recognit..

[30] Gong M., Zhou Z., Ma J. (2012). Change detection in synthetic aperture radar images based on image fusion and fuzzy clustering. IEEE Trans. Image Process..

[31] Lee J.S., Hoppel K.W., Mango S.A., Miller A.R. (1994). Intensity and phase statistics of multilook polarimetric and interferometric SAR imagery. IEEE Trans. Geosci. Remote Sens..

[32] Joughin I.R., Winebrenner D.P., Percival D.B. (1994). Probability density functions for multilook polarimetric signatures. IEEE Trans. Geosci. Remote Sens..

[33] Bazi Y., Bruzzone L., Melgani F. (2005). An unsupervised approach based on the generalized Gaussian model to automatic change detection in multitemporal SAR images. IEEE Trans. Geosci. Remote Sens..

[34] Bronshtein I.N., Semendyayev K.A., Musiol G., Muehlig H. (2007). Handbook of Mathematics.

[35] Bujor F., Trouve E., Valet L., Nicolas J.M., Rudant J.P. (2004). Application of log-cumulants to the detection of spatiotemporal discontinuities in multitemporal SAR images. IEEE Trans. Geosci. Remote Sens..

[36] Dekker R.J. (1998). Speckle filtering in satellite SAR change detection imagery. Int. J. Remote Sens..

[37] Sharifi K., Leon-Garcia A. (1995). Estimation of shape parameter for generalized Gaussian distributions in subband decomposition of video. IEEE Trans. Circuits Syst. Video Technol..

[38] Niehsen W. (1999). Generalized Gaussian modeling of correlated signal sources. IEEE Trans. Signal Process..

[39] Inglada J., Mercier G. (2007). A new statistical similarity measure for change detection in multitemporal SAR images and its extension to multiscale change analysis. IEEE Trans. Geosci. Remote Sens..

[40] Kullback S., Leibler R.A. (1951). On information and sufficiency. Ann. Math. Stat..

